# Effects of Nickel and Iron Content on the Microstructures and Mechanical Properties of Cemented Carbide with Coarse and Fine-Grained Heterostructures

**DOI:** 10.3390/ma18092045

**Published:** 2025-04-30

**Authors:** Shuzhong Yang, Nan Ye, Mingxian Zhang, Yaru Zhu, Chenxin Zhang, Wentan Zhu, Fan Zhang, Jiafa Jiang, Jiancheng Tang

**Affiliations:** 1 School of Physics and Materials Science, Nanchang University, Nanchang 330031, China; yangsz1989@163.com (S.Y.); 19834924900@163.com (Y.Z.); 2 International Institute for Materials Innovation, Nanchang University, Nanchang 330031, China; yenan@ncu.edu.cn (N.Y.); zhuwentan@ncu.edu.cn (W.Z.); tangjiancheng@ncu.edu.cn (J.T.); 3 China Nerin Engineering Company Limited, Nanchang 330031, China; zhangchenxin@nerin.com; 4 Ganzhou Nonferrous Metallurgy Research Institute Company Limited, Ganzhou 341099, China; zf_419@163.com (F.Z.); jiangjiafa01@163.com (J.J.)

**Keywords:** cemented carbides, heterogeneous structure, strength, toughness, toughening mechanism

## Abstract

Cemented carbides are composite materials that combine both structural and functional properties. However, the inherent trade-off between strength and toughness presents a significant challenge in fully leveraging the synergistic potential of these dual-phase materials. In this study, cemented carbides with coarse and fine-grained heterogeneous structure were fabricated. The effects of nickel (Ni) and iron (Fe) content on the microstructures and mechanical properties of these heterogeneously structured cemented carbides were systematically investigated. Microstructural analysis revealed that the fine-grained granules are uniformly embedded in the coarse-grained region, forming a typical network-like mixed-grain structure. The introduction of the heterogeneous structure enables cemented carbides to achieve a remarkable balance of high strength and toughness. Specifically, the materials exhibit optimal strength–toughness matching with a transverse rupture strength of 2949 MPa, a fracture toughness of 23.65 MPa·m^−1/2^, and a hardness of 1430 HV when the proportion of Ni and Fe content reaches 4.2 wt.%. The toughening mechanism is primarily attributed to the increased volume fraction and stabilized dimensions of CoNiFe binder phases, which promote interfacial decohesion at WC/WC and WC/binder boundaries while suppressing transgranular fracture. These mechanisms collectively contribute to enhanced toughening and crack propagation resistance. This study establishes foundational insights into achieving a synergistic combination of strength and toughness in cemented carbides.

## 1. Introduction

Cemented carbides, fabricated through the powder metallurgy process, are typically composed of refractory metals (such as tungsten carbide and titanium carbide) and bonding metals (e.g., cobalt and nickel). These materials are renowned for their high hardness, exceptional strength, excellent wear resistance, good corrosion resistance, and remarkable red hardness [1,2,3]. Significantly harder than conventional steels, cemented carbides maintain their dimensional accuracy over prolonged periods, which makes them indispensable in the manufacture of metal-cutting tools, including turning tools and milling cutters. They are also extensively used in mining equipment, such as drills and cut-off teeth, where they effectively break down rocks and other hard materials. However, the rapid development of the manufacturing industry and the enhanced overall performance of the machined materials present a critical challenge to the achievement of both high strength and toughness in traditional cemented carbides [4]. In this context, developing methodologies to enhance toughness without compromising strength has emerged as a focal point of study.

The classical tungsten carbide–cobalt (WC-Co) cemented carbides exhibit exceptional hardness, superior cutting properties, excellent grinding capability, and good wear resistance under the operational conditions [5,6]. For instance, WC-Co cemented carbide tools exhibit high machining efficiency and quality and can continuously cut hard metal materials for extended periods without dulling the cutting edge. Co acts as a binder phase, facilitating the cohesion of numerous WC particles into a cohesive alloy structure. When the external forces are applied, the Co binder can absorb and distribute the stresses through its own plastic deformation, thereby preventing the shedding or fracturing of WC particles due to stress concentration. However, the constraints imposed by phase defects in the cobalt binder can lead to brittle fracture when cemented carbides are subjected to excessive impact or abrupt stress changes. Hence, it is significant to enhance the strength and toughness of cemented carbides.

To date, various methods have been proposed to enhance the strength and toughness of cemented carbides. One of the most popular strategies is alloying, which involves refining grain size and controlling interfacial adaptability to enhance the overall performance of cemented carbide tool materials [7,8,9,10,11,12,13,14,15,16]. The incorporation of specific alloying elements into cemented carbides can significantly improve their properties. For instance, the addition of minor quantities of elements such as Cr, V, and Ti can result in the formation of special compound phases or solid solutions with WC-Co, thereby enhancing the strength and toughness of cemented carbides. It had also been reported that the certain strength–toughening effects were achieved by introducing alloying elements including Zr [7], ZrO_2_ [8], TiC [9], Al_2_O_3_ [10], Y_2_O_3_-ZrO_2_ [11], Cr_2_(C, N) [12], and so on. Additionally, high entropy alloys, such as CoCrNiCuFe [13], CoCrFeNiAl [14], CoCrFeNi [15], and AlCuMnMoTi [16], have been employed to replace the Co binder phase, aiming to enhance the overall performance of cemented carbides. However, the high price of incorporating reinforcing phases increases the production cost of cemented carbides. Moreover, the lack of maturity in preparation technology poses challenges for large-scale industrial production, hindering the attainment of desirable mechanical properties in cemented carbide products [12].

Inspired by nature, a revolutionary material known as heterostructured materials, characterized by heterogeneous regions with differences in mechanical or physical properties, has been introduced into the design of cemented carbides [17]. These novel heterogeneous structures include laminated, bimodal, multimodal, harmonic (Core-Shell), and gradient structures, as well as coarse and fine-grained structures. Their unique structure helps to break the typical trade-off relationship between strength and toughness. The development of heterogeneous structures in WC-Co alloys, combined with the influencing content of alloying, may represent a promising approach to simultaneously enhance both strength and toughness.

In this study, cemented carbides with a heterogeneous structure were prepared by designing a novel binder composed of Co, Ni, and Fe elements. The effects of Ni and Fe content on the evolution of the microstructures and mechanical properties were systematically investigated.

## 2. Materials and Methods

### 2.1. Materials Preparation

**Figure 1:**
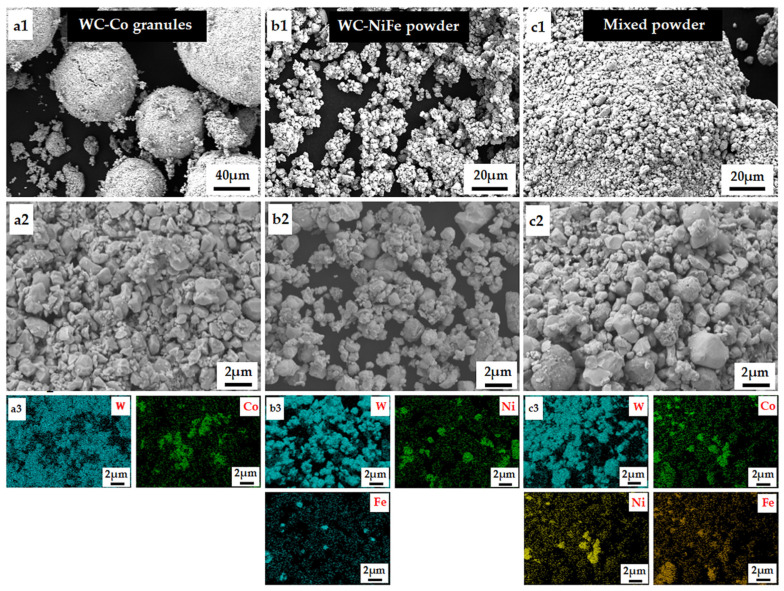
SEM images and corresponding elemental distribution maps of raw powders: (**a1**–**a3**) WC-Co granules, (**b1**–**b3**) WC-NiFe powders, (**c1**–**c3**) mixed powders.

The raw materials used in the experiments included commercial WC powder, Ni powder, Fe powder, and WC-6Co granules. Their scanning electron microscope (SEM) images are presented in Figure 1. The WC-6Co granules were supplied by Jiangxi Jiangwu Cemented Carbide Co., Ltd. (Yichun, China), and exhibited a regular spherical morphology with an average particle size ranging from 80 to 120 μm (Figure 1(a1–a3)). Based on the high-magnification SEM images and corresponding elemental distribution maps, Co and W elements were observed to be uniformly distributed within the granules, and the average grain size of WC was approximately 2 μm. The SEM images of the WC-NiFe mixed powders are shown in Figure 1(b1–b3), where the average particle sizes of WC, Ni, and Fe were selected to be 7 μm, 3~5 μm, and 3~5 μm, respectively.

The Ni/Fe ratio was determined to be 3:1 in this study. The powders were weighed according to the designed composition and wet-milled in a roller ball mill with 2 wt.% paraffin added as a plasticizer. The specific ball milling parameters were as follows: the weight ratio of balls to material was 5:1, the grinding time was 40 h, the diameter of the balls was 6 mm, and the alcohol content with 99.9% purity was 400 mL/kg. Subsequently, the milled powders were dried in a vacuum drying oven to obtain mixed WC-NiFe powders.

The cemented carbides with a heterogeneous structure were prepared by adjusting the proportion of added Ni and Fe elements. The specific parameters and the corresponding alloy compositions are presented in Table 1. The designed proportion of the added Ni and Fe powder granules were 2.4 wt.%, 3 wt.%, 3.6 wt.%, and 4.2 wt.%. The baseline cobalt content (4.2 wt.%) serves as the reference for equivalent metallic binder phase replacement. The chosen percentages represent progressive substitution levels from 36 wt.% to 50 wt.% of the original Co content, enabling the systematic investigation of the replacement effects of Fe and Ni. The WC-Co granules and WC-NiFe powders were weighed and mixed in an agitator. The agitation rate was set at 168 rpm, the temperature was 60 °C, and the agitating time was 3 h.

The SEM images of the mixed powders are shown in Figure 1(c1–c3), where the granules adhered to the WC-NiFe powders maintained their regular spherical morphologies. The mixed powders were pressed into rectangular blanks (20 mm in length, 6.5 mm in width, and 5.25 mm in height for microstructural examination; 40 mm in length, 7.4 mm in width and 4.8 mm in height for the fracture toughness test) at an axial pressure of 150 MPa for 15 s, then the specimens underwent thermal debinding in a hydrogen atmosphere at 500 °C for 2 h to remove the paraffin and oxygen. Subsequently, they were further heated to 1410 °C and held for 1 h under a pressure of 5 MPa in an argon atmosphere.

### 2.2. Microstructure Characterization

**Figure 2:**
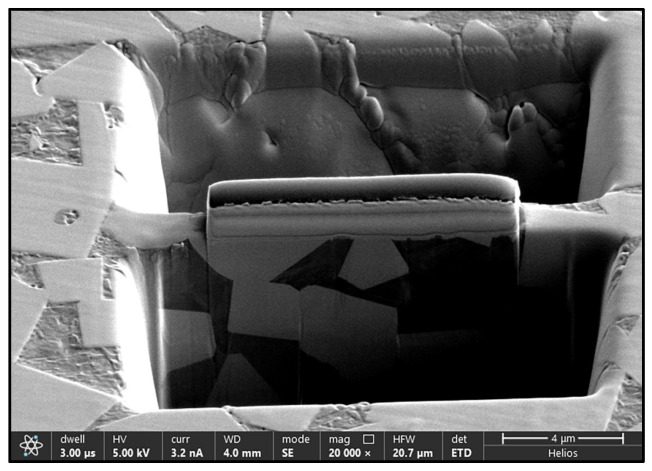
TEM specimen prepared by FIB technology.

The specimens were first machined by electrical discharge machining for the microstructural characterization. The surfaces of the specimens were grinded with 2000 grit abrasive paper and then polished with a 0.5 μm diamond polishing spray. The microstructure and elemental composition were characterized using a field emission scanning electron microscope (SEM, FEI Inspect F50, Hillsboro, OR, USA) equipped with an Energy Dispersive Spectrometer (Oxford X-Max50, Oxfordshire, UK). The phases of the alloys were determined using X-ray diffraction (XRD, Bruker Advance D8, Billerica, MA, USA) with Cu Kα radiation (λ = 1.5406 Å) at a scanning angle ranging from 20° to 90°, and a scan speed of 1°/min and a scan step of 0.02 were used for the XRD measurements. The microstructures of the WC/γ-(Co, Ni, Fe) specimens’ interface were characterized by using a focused ion beam (FIB) and transmission electron microscopy (TEM, Thermo Scientific Spectra 300S, Waltham, MA, USA). An FIB system was utilized to trench a cross-section of the interfaces. As seen in Figure 2, a 5 kV Ga+ ion beam with a beam current of 3.2 nA was used to produce a 50 nm thick lamella. High-angle annular dark field imaging (HAADF) and energy dispersive spectroscopy (EDS) mapping were conducted to investigate the phase composition and elemental distribution of the specimens.

### 2.3. Mechanical Properties Testing

**Figure 3:**
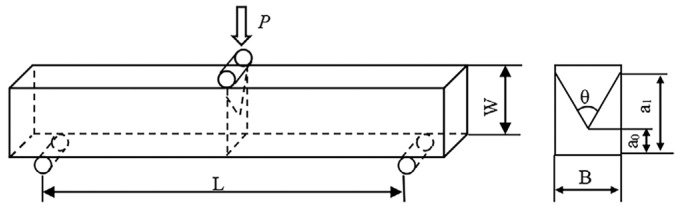
The dimensions of the specimen for the fracture toughness test (L = 40 mm; W = 7.4 mm; B = 4.8 mm; θ = 60°; a_0_ = 2.74 mm; a_1_ = 3.08 mm).

The transverse rupture strength (TRS) of the specimens was measured by using a three-point bending strength testing machine (CMT5105, Sansi, Hangzhou, China) with a longitudinal spacing of 14.5 mm and a load rate of 4 mm/min. Formula (1) was employed to evaluate the TRS of the specimen in the three-point bending test [18].

(1) $$R=\frac{3FL}{2bh^{2}}$$

where *R* is the transverse rupture strength (MPa), *F* is the maximum applied load (N), *L* is the spacing between the two pivots (mm), *b* is the width of the test piece (mm), and *h* is the height of the test piece (mm). The relative densities of all the alloys were measured using the Archimedean method. The hardness was measured by a Vickers hardness tester with the indentation loading force of 30 Kgf and a dwell time of 15 s. The fracture toughness of the alloy was tested using the V-notch method, and the geometry of the specimen, along with its dimensions, are shown in Figure 3 [19].

The fracture toughness (*K_IC_*) was calculated by the indentation crack method according to Formula (2) [20].

(2) $$K_{IC}=0.0028\sqrt{\frac{HV\cdot P}{\sum L}}$$

where *K_IC_*, *P*, *L*, *HV* represent the facture toughness (MPa·m^−1/2^), indentation load (N/mm^2^), crack propagation length (mm), and Vickers hardness value, respectively. At least five specimens were measured to ensure the accuracy of the mechanical properties.

## 3. Results and Discussion

### 3.1. Effects of Ni and Fe Content on the Microstructures

**Figure 4:**
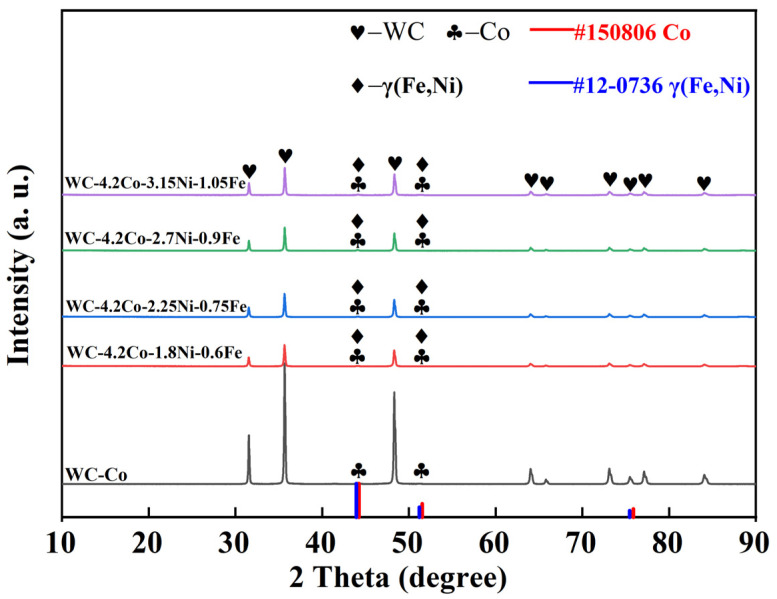
XRD patterns of conventional WC-Co cemented carbides and heterogeneously structured WC-Co-Ni-Fe cemented carbides.

The XRD diffraction spectrum of conventional WC-Co cemented carbides and heterogeneously structured WC-Co-Ni-Fe cemented carbides is displayed in Figure 4. For the conventional tungsten carbide, only diffraction peaks of WC and Co are detected. It can be seen that the alloy is composed of WC and Co phases. For the HS specimen with the addition of Ni and Fe, additional diffraction peaks of the γ-(Ni, Fe) phase are detected. However, the diffraction peaks of the γ-Ni (Fe) and Co phases are very close, as indicated by the corresponding PDF card (#15-0806 for Co, #12-0736 for γ-(Ni, Fe)), making it difficult to distinguish the composition of the alloys with heterogeneous structures based only on XRD results. Therefore, in-depth tests are necessary to study the existence of phases and the distribution of elements.

**Figure 5:**
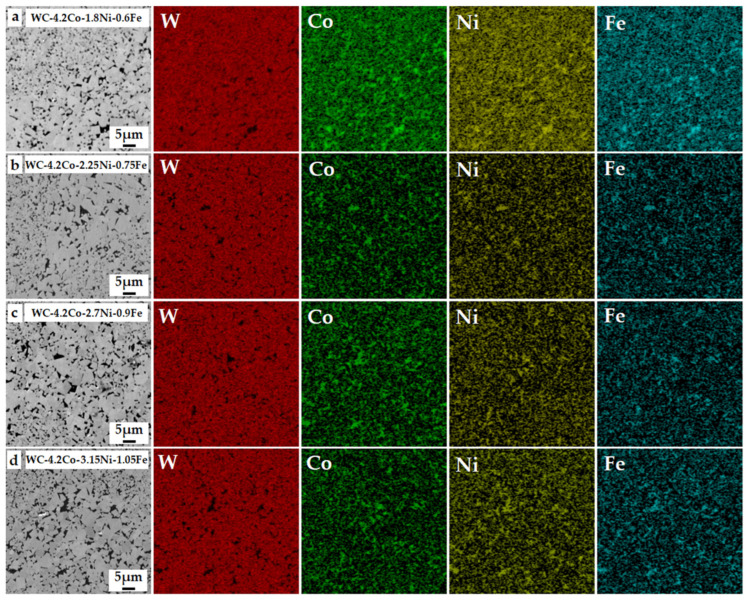
The BSE-SEM images and corresponding EDS results of the cemented carbides with Ni and Fe contents of (**a**) 2.4 wt.%, (**b**) 3 wt.%, (**c**) 3.6 wt.%, (**d**) 4.2 wt.%.

The BSE-SEM images and corresponding EDS results of the heterogeneously structured WC-Co-Ni-Fe cemented carbides are presented in Figure 5. Microstructural evolution analysis indicates that cemented carbides consist of the gray hard WC phases and the black binder phases. The binder phases are uniformly distributed in the WC matrix, showing a homogeneous distribution without significant elemental segregation. The EDS results indicate that the Co, Ni, and Fe elements are distributed within the same regions of the binder phase, suggesting that liquid sintering occurred.

**Figure 6:**
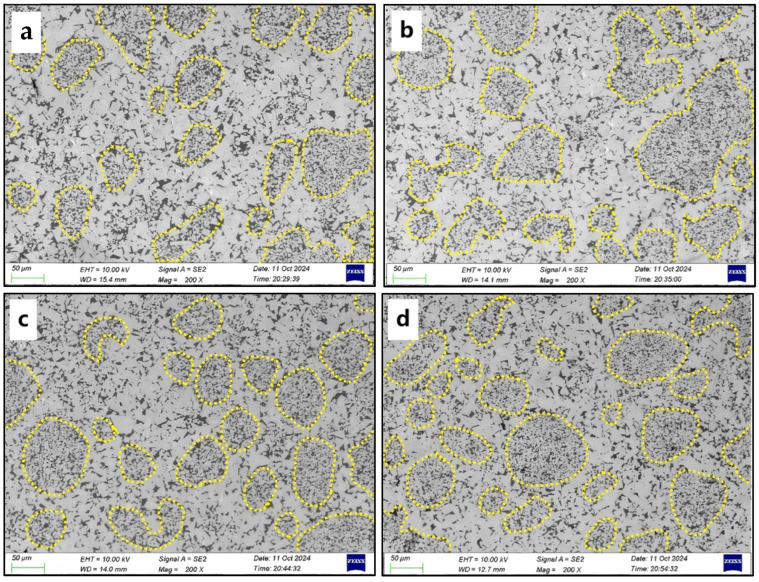
BSE-SEM images of the WC-CoFeNi cemented carbides with additional Ni and Fe contents of (**a**) 2.4 wt.%, (**b**) 3 wt.%, (**c**) 3.6 wt.%, (**d**) 4.2 wt.%.

**Figure 7:**
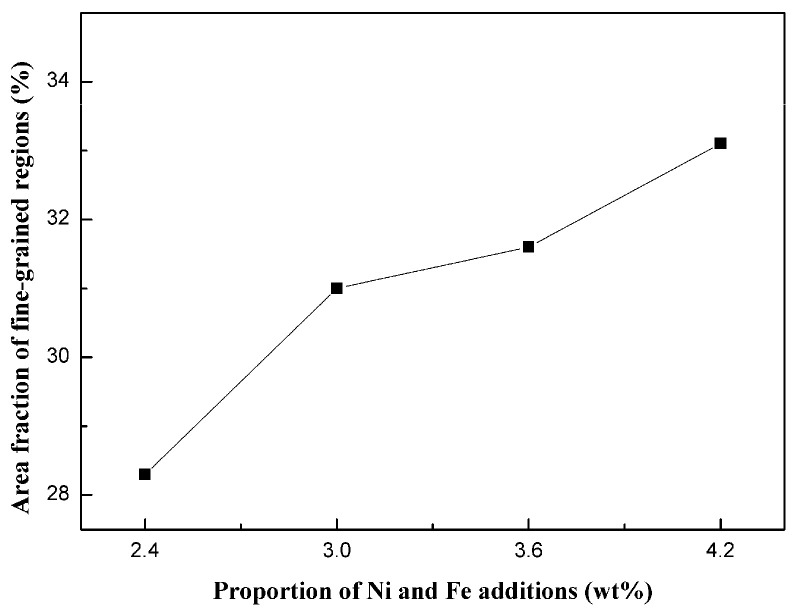
Area fraction of fine-grained regions of WC-CoFeNi cemented carbides.

The microstructure evolution of the cemented carbides with Ni and Fe contents of 2.4 wt.%, 3 wt.%, 3.6 wt.%, and 4.2 wt.% is shown in Figure 6. The microstructure of the specimens is divided into the fine-grained region (circled by the yellow dotted lines, corresponding to WC-Co granules) and the coarse-grained region (corresponding to WC-NiFe powders), where the fine grains are uniformly embedded in the coarse-grained region, presenting a typical network mixed-grain structure. The area fraction of fine-grained regions of the WC-CoFeNi cemented carbides with varying Ni and Fe contents is shown in Figure 7. It can be seen that the total area within the dotted line increases with increasing Ni and Fe content. This indicates that the volume fraction of the fine-grained regions and the overall content of the binder phase increase. When the proportion of Ni and Fe content reaches 4.2 wt.%, the shape of the fine-grained regions becomes nearly circular, and both the number and the area of the fine-grained regions reach their maximum.

**Figure 8:**
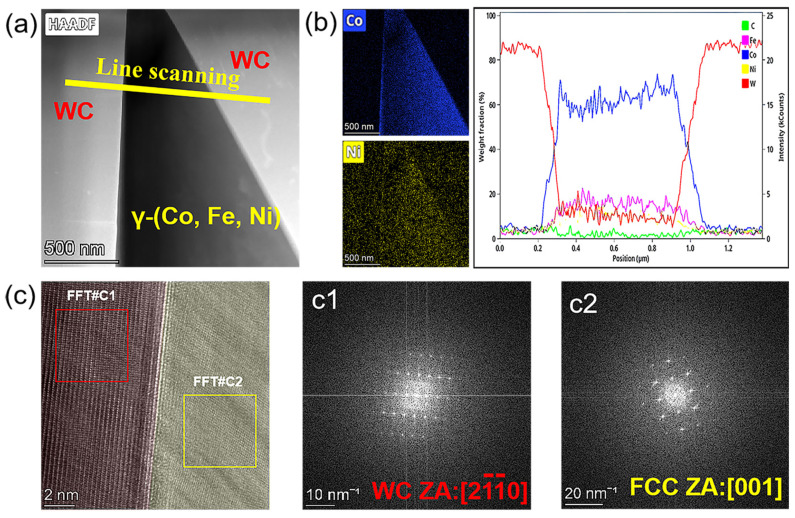
TEM images of the WC-4.2Co-1.8Ni-0.6Fe cemented carbide: (**a**) the microstructure of the WC/γ-(Co, Ni, Fe) interface, (**b**) the elemental distribution maps and line scanning results of the interface, and (**c** (**c1**,**c2**)) the FFT diffractogram image.

The HAADF images of the WC/γ-(Co, Ni, Fe) interface taken by the TEM are shown in Figure 8. The elemental mapping distribution and corresponding elemental scanning results are presented in Figure 8a,b, which confirm that the dark area is the binder phase whose boundaries are adjacent to the WC phase. The elemental scanning results indicate that the Fe, Ni, and Co elements are uniformly distributed in the binder during the liquid sintering process. It can be further evidenced by the fast Fourier transformation (FFT) diffractogram image in Figure 8c, which shows that the zone belongs to the WC phase, and the [001] zone belongs to the γ-(Co, Ni, Fe) phase, respectively.

**Figure 9:**
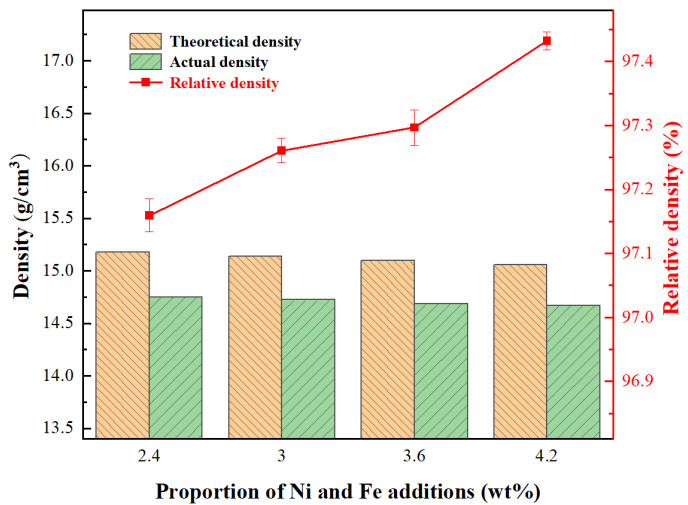
Theoretical density, actual density, and relative density of WC-CoFeNi cemented carbides.

Density and relative density are important indicators for measuring the sintering densification of the alloys. Generally, an increase in a material’s relative density signifies fewer internal flaws and holes. Figure 9 shows the theoretical density, actual density, and relative density of the WC-CoFeNi cemented carbides with varying Ni and Fe content. It can be seen that the actual density of the cemented carbides decreases with the increase in Ni and Fe content. Since the theoretical density of cemented carbides also decreases with the addition of Ni and Fe due to their low density, the relative density of cemented carbides displays an ascending trend, indicating that the structure of the alloy becomes denser. When the proportion of Ni and Fe content is 2.4 wt.%, the relative density of the alloys is 97.15%. As the proportion rises to 4.2 wt.%, the specimens achieve a maximum relative density of 97.43%. This suggests that the addition of Ni and Fe enhances the sintering compactness of alloys.

The liquid sintering process contributes to achieving a high degree of densification and a uniform distribution of the cemented carbide base on the dissolution–precipitation mechanism. To study the effects of Ni and Fe content on the density of the material, the calculation formula is shown in Formula (3) [21].

(3) $$D=D_{0}+\frac{d_{b}}{d_{a}}exp\left[\frac{-0.17T_{m}\left(16+K_{0}\right)}{T}\right]$$

where *D* is the diffusion coefficient, *D*_0_ is the frequency factor, *d_a_* and *d_b_* are the Goldschmidt radii of the solvent metal and non-metal atoms. *T* is the holding temperature, *T_m_* is the melting point of the binder phase, *K*_0_ is the valence state of the atom [21,22]. It can be inferred from the formula that the diffusion coefficient and the melting point of the binder phase are inversely proportional. The sintering density of WC-CoFeNi cemented carbides is influenced by both the melting point and the content of the liquid phase. It is known from the phase diagram of the CoNiFe alloy that the melting point of the alloy is significantly lower than that of pure Co. Therefore, the melting point of the binder phase decreases with the addition of Ni and Fe elements, resulting in an increase in the diffusion coefficient. This enhances the flow and rearrangement of the particles, leading to a more uniform distribution of the binder phase within the cemented carbides. Compared to using pure Co as the binder, adding Ni and Fe elements to the binder results in a higher liquid phase proportion and a higher sintering density at the same sintering temperature. Finally, the densification of WC-CoNiFe cemented carbides primarily occurs during the sintering process.

According to the Ostwald ripening process, the fine WC grains tend to be dissolved preferentially in the liquid bonder phase, which can easily prompt the coarsening of the coarse WC grains due to the reprecipitation process [23]. Since W exhibits high solubility in Co, a significant amount of W in fine WC grains dissolves into the binder phase. When the proportion of Ni and Fe content increases, the dissolution and reprecipitation of fine WC grains are reduced due to their lower solubility in the binder. During sintering, the CoNiFe binder suppressed the interfacial redeposition of tungsten (W) and carbon (C) atoms from the supersaturated binder phase onto the surfaces of coarse WC grains. Thus, the volume fraction of fine WC grains markedly increases with the increase of Ni and Fe elements added in the cemented carbides.

Moreover, the addition of Fe and Ni elements is beneficial for increasing the roundness of the WC grains. WC belongs to the simple hexagonal system (type P-6m2), with each unit cell containing one W atom and one C atom. The C atom occupies an asymmetric position (1/3, 2/3, 1/2) in the unit crystal system [24,25,26]. Schubert demonstrated that the asymmetric occupancy of C atoms led to the unbalanced growth of the WC crystal, resulting in the formation of trigonal prisms, triangular plates, and hexagonal plates of WC [24]. The presence of Fe and Ni atoms reduced the interfacial energy between the WC and binder phase [27], which inhibited the flattening tendency of WC grains. As a result, the Co-Fe-Ni binder system promoted the formation of nearly spherical WC grains due to the strong inhibitory effect of Fe and Ni elements on unbalanced grain growth.

### 3.2. Effects of Ni and Fe Content on Mechanical Behavior

**Figure 10:**
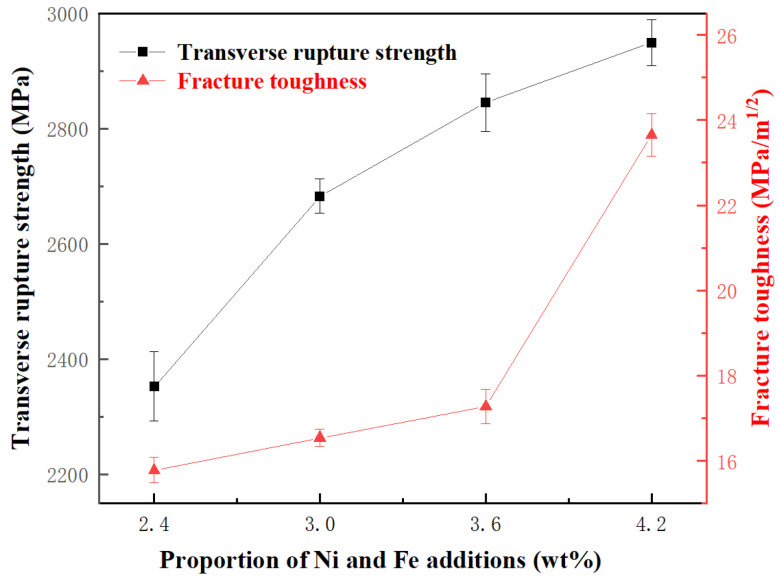
Rupture strength and fracture toughness of WC-CoFeNi cemented carbides.

**Figure 11:**
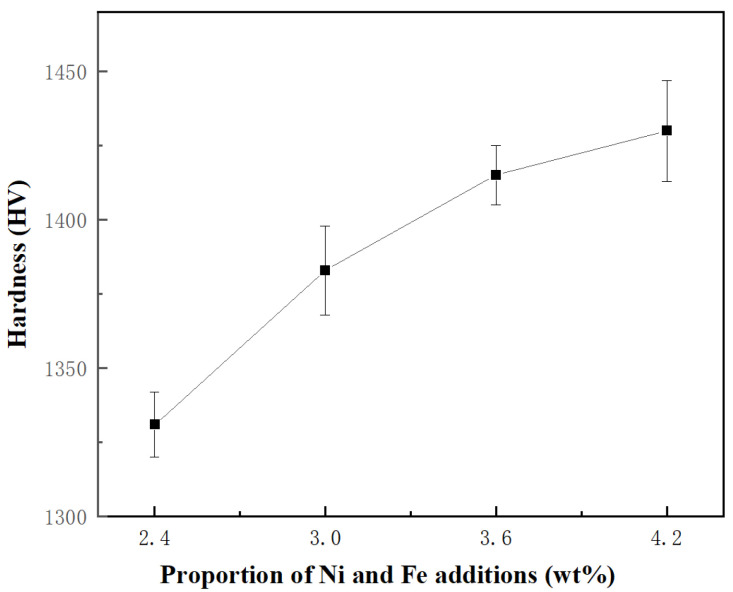
The hardness of WC-CoFeNi cemented carbides.

The transverse rupture strength and the fracture toughness of the specimens were measured in this study. The specimens underwent four separate lines of testing, and the average of these values was taken as the performance value. As shown in Figure 10 and Figure 11, the mechanical properties of the specimens are significantly influenced by the proportion of Ni and Fe content. The transverse rupture strength, fracture toughness, and the hardness of WC-CoNiFe cemented carbides gradually increase and reach maximum values with the increase in the proportion of Ni and Fe content. When the proportion of Ni and Fe content is 2.4 wt.%, the specimens exhibit the lowest rupture strength of 2352 MPa, the lowest fracture toughness of 15.78 MPa·m^−1/2^, and the lowest hardness of 1332 HV. As the proportion increases to 3 wt.%, the rupture strength of the specimen increases to 2683 MPa, the fracture toughness improves to 16.54 MPa·m^−1/2^, and the hardness grows to 1384 HV. When the proportion reaches 4.2 wt.%, the specimens achieve the maximum rupture strength of 2949 MPa, the maximum fracture toughness of 23.65 MPa·m^−1/2^, and the maximum hardness of 1430 HV.

As the melting point of the binder phase decreases with the increasing content of Ni and Fe, the diffusion coefficient and the proportion of the liquid phase increase. This leads to a higher sintering density of the cemented carbides. The phenomenon can be attributed to the filling of spaces between WC particles by the increased liquid binder phase, which promotes the densification of the cemented carbides. Furthermore, the relationship between density and hardness can be described by Formula (4) [28].

(4) $$H=K\times d^{-a}\times e^{-b\varepsilon }$$

where *H* represents the hardness of the cemented carbides; *K*, *a*, and *b* are constants; *ε* denotes the porosity; and *d* is the grain size of WC. This formula signifies an inverse relationship between porosity and hardness, as well as a direct relationship between WC grain size and hardness. As depicted in Figure 7 and Figure 9, both the area fraction of fine-grained regions and the relative density show a monotonic increase with higher Ni and Fe contents. Specifically, the cemented carbides with 4.2 wt.% of Ni and Fe exhibit peak hardness values due to their refined microstructure and high relative density. This highlights the crucial role of Ni and Fe additions in inhibiting grain growth through liquid-phase sintering and in aiding pore elimination.

**Figure 12:**
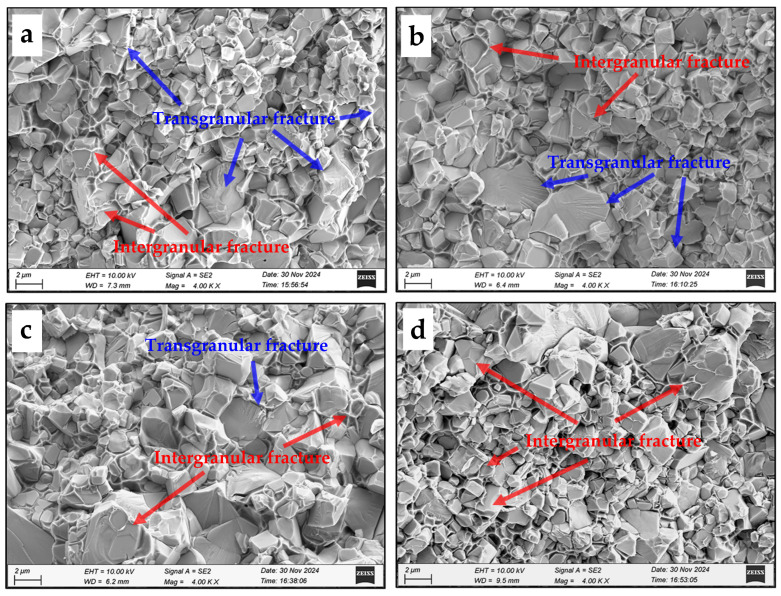
Fracture morphology in the cemented carbides with additional Ni and Fe contents of (**a**) 2.4 wt.%, (**b**) 3 wt.%, (**c**) 3.6 wt.%, (**d**) 4.2 wt.%.

**Figure 13:**
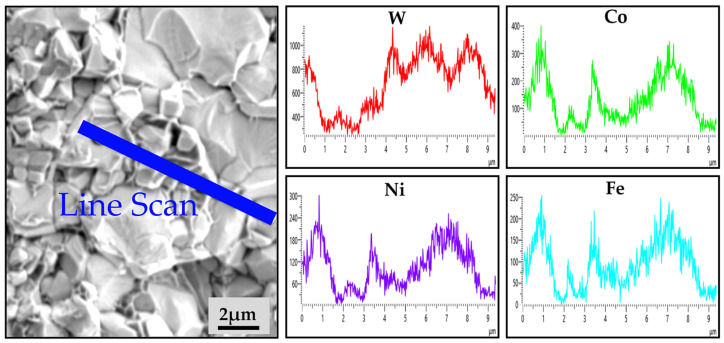
SEM-EDS line scan of cemented carbides with an additional Ni and Fe content of 2.4 wt.%.

The fracture morphology in the cemented carbides with varying Ni and Fe content is illustrated in Figure 12. The SEM-EDS line scan of cemented carbides with an additional Ni and Fe content of 2.4 wt.% is shown in Figure 13. It reveals that the Co, Ni, and Fe elements are distributed within the same regions of the binder phase. Furthermore, there are two modes of crack propagation, i.e., intergranular fracture and transgranular fracture. The fracture surface in Figure 12a demonstrates a mixed morphology containing both intergranular and transgranular fracture types. Transgranular fracture occurs when the compressive stress acting on the WC grains is sufficient to induce brittle fracture. The coarser WC grains tend to generate larger compressive stress, resulting in prominent transgranular cleavage features. Figure 12b–d indicate a progressive transition in the failure mechanism with increasing Ni and Fe content. The enhanced volume fraction and dimensional stability of CoNiFe binder phases promote the interfacial decohesion at WC/WC and WC/binder boundaries, thereby suppressing transgranular fracture while facilitating intergranular crack propagation.

The strength of cemented carbides is determined by the interfacial bonding strength and stability, which are further influenced by the electronic structure and bonding behavior at the WC/CoNiFe interface. Since Fe, Ni, Co, and W atoms can bond with each other, the interface spacings between Fe and W atoms are smaller than those between Co and W atoms [29]. As the concentration of Fe atoms increases, stronger interactions, tighter chemical bonds, and a better match in electronic structure were achieved. Therefore, when strong covalent bonding with high interfacial binding energy is established at the WC/CoNiFe interface, the solid solution strengthening of the binder is enhanced. This effectively increases the energy required for crack propagation and inhibits the initiation and growth of microcracks. As a result, the strength of the cemented carbides increased with increasing Ni and Fe content.

The fracture toughness of the specimens also increases with the increase in Fe and Ni additions. This enhancement can be primarily attributed to the increased amount of the binder phase and the morphology of the WC grains. On one hand, the binder phase with a face-centered cubic (FCC) structure exhibits superior plasticity due to its greater number of slip systems. The increase in the FCC binder phase enhances the fracture toughness of WC-CoNiFe. On the other hand, the shape of the WC grains becomes nearly spherical due to the strong inhibition effect of the Fe and Ni binder on unbalanced growth. The increased proportion of nearly spherical WC grains alleviates the local stress concentration [30,31], making crack propagation more difficult and thereby enhancing the fracture toughness of cemented carbides. Finally, the improved fracture toughness of WC-CoNiFe cemented carbides can be explained by the superior plasticity of binder phase and the optimized morphology of the WC grains.

## 4. Conclusions

In this work, the effects of Ni and Fe content on the microstructure and mechanical properties of WC-CoNiFe cemented carbides were investigated. The main findings are as follows:

(1) The cemented carbides with a heterogeneous structure consist of a fine-grained region (corresponding to WC-Co granules) and a coarse-grained region (corresponding to WC-NiFe powders). Fine-grained granules are uniformly embedded in the coarse-grained region, forming a typical network mixed-grain structure.
(2) As the Ni and Fe content increase from 2.4 to 4.2 wt.%, the actual density of cemented carbides decreases, while the average relative density increases from 97.15% to 97.43%. The addition of Ni and Fe elements contributes to reducing the melting point and prompting the diffusion coefficient of cemented carbides, which can be attributed to liquid phase sintering.
(3) As the proportion of Ni and Fe content increased, the average transverse rupture strength increased from 2352 to 2949 MPa, the average fracture toughness increased from 15.78 to 23.65 MPa·m^−1/2^, and the average hardness increased from 1332 to 1430 HV. The enhanced volume fraction and dimensional stability of CoNiFe binder phases promote the interfacial decohesion at WC/WC and WC/binder boundaries, suppressing transgranular fracture while facilitating intergranular crack propagation.

## Figures and Tables

**Table 1:** Compositions of the designed cemented carbides.

Designing Cemented Carbides	Proportion				Proportion of Ni and Fe Content	Alloy Compositions	Theoretical Density (g/cm^3^)
	WC-6Co	WC-NiFe					
		WC	Ni	Fe			
H0	100	0	0	0	0	94WC-6Co	15.23
H1	70	27.6	1.8	0.6	2.4 wt.%	WC-4.2Co-1.8Ni-0.6Fe	15.18
H2	70	27	2.25	0.75	3 wt.%	WC-4.2Co-2.25Ni-0.75Fe	15.14
H3	70	26.4	2.7	0.9	3.6 wt.%	WC-4.2Co-2.7Ni-0.9Fe	15.10
H4	70	25.8	3.15	1.05	4.2 wt.%	WC-4.2Co-3.15Ni-1.05Fe	15.06

## Data Availability

The original contributions presented in this study are included in the article. Further inquiries can be directed to the corresponding author.
